# Shifting from control to elimination: analysis of malaria epidemiological characteristics in Tengchong County around China-Myanmar border, 2005-2014

**DOI:** 10.1186/s12936-016-1089-9

**Published:** 2016-01-28

**Authors:** Shengguo Li, Shouqin Yin, Jiazhi Wang, Xishang Li, Jun Feng

**Affiliations:** National Institute of Parasitic Diseases, Chinese Center for Disease Control and Prevention; Key Laboratory of Parasite and Vector Biology, Ministry of Health; WHO Collaborating Centre for Tropical Diseases, National Center for International Research on Tropical Diseases, 200025 Shanghai, China; Tengchong County Centers for Disease Control and Prevention, No. 51 Guanghua village, Tiancheng district, 679100 Tengchong, Yunnan Province China

**Keywords:** Malaria, Epidemiology, Tengchong County, Elimination, China-Myanmar border

## Abstract

**Background:**

Tengchong County experienced a decreasing malaria prevalence period in 2005–2014 but the factors contributing to the trend are unclear. Herein, the malaria epidemiological data in years of 2005–2014 were collected and analysed, in order to provide evidence for subsequent effective strategic planning of malaria elimination that may be referenced by other counties with the similar elimination programmes along the China-Myanmar border.

**Methods:**

A retrospective study was conducted to explore malaria-endemic characteristics in years 2005–2014 in Tengchong County. All individual cases from a web-based reporting system were reviewed and analysed. Local infections and imported cases were obtained from an annual reporting system.

**Results:**

In total, 8321 confirmed malaria cases were recorded in this period, and 91.5 % of them were reported during 2005–2010. *Plasmodium vivax* was the major species (n = 5867, 70.5 %). Most cases (92.9 %) were found in males, mainly in the age group 30–34 years. Only five deaths resulting from *Plasmodium falciparum* were reported, of which three occurred in 2005. The cases were mainly reported in the townships of Wuhe (18.5 %), Mangbang (12.8 %) and Gudong (9.3 %). In addition, 147 local malaria (1.8 %) and 8174 imported malaria (98.2 %) were observed during 2005–2014. However, the proportion of imported malaria was more than 95 % all the time and no local transmission has been observed since 2013. Moreover, Myanmar was the main imported source, with 716 cases (94.6 %, 716/757) from Myanmar in 2011–2014.

**Conclusions:**

Tengchong County has made achievements in controlling malaria, with incidence at historically its lowest level. However, imported malaria has increased and poses a great threat to malaria elimination. To achieve the elimination goal and prevent the re-introduction of malaria, surveillance systems need to be well planned and managed to ensure timely case detection and prompt response targeted to the mobile and migrate population at elimination stage.

## Background

Malaria is caused by one or more of the five species of *Plasmodium* via the bite of infected female *Anopheles* mosquitoes. An estimated 214 million confirmed cases were reported from 75 countries and territories in 2014, with 438,000 deaths [1]. China has made obvious achievements in controlling locally transmitted malaria over the past decades and incidence was down to historically low levels in 2009 [2, 3]. China initiated the National Malaria Elimination Programme (NMEP) in 2010, which aimed to eliminate indigenous malaria, except for border areas, by 2015, and to completely eliminate nationwide by 2020 [4].

**Fig. 1:**
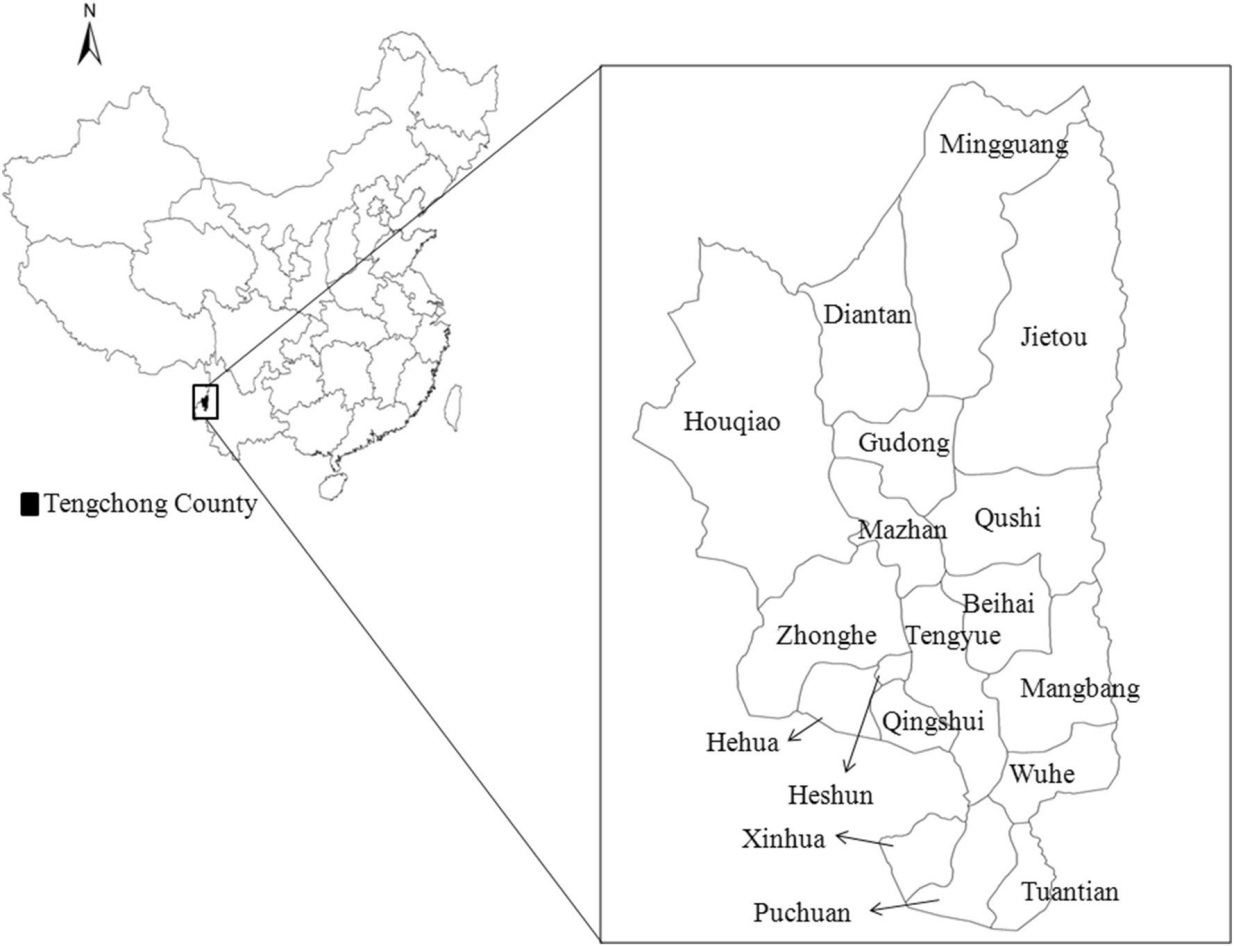
Location of Tengchong County in China. The names of 18 townships are labelled on the right

Despite this, malaria along the border regions is still a great challenge to the elimination process. For example, although there was a 99.1 % decrease in local cases reported from 2005 (n = 5119) to 2014 (n = 47) in 18 counties along the China-Myanmar border, this still accounts for 82.5 % of total local cases throughout the whole country [5]. Tengchong County is one of the 18 border counties with a total population of 6,681,000 and a border line of 148 km, located in the southwest of Yunnan Province. It has 18 townships of which three are bounded by Kachin State Special Region-1, Myanmar (Fig. 1). Tengchong County was an endemic area, prone to large outbreaks and classified a malaria-unstable region [4]. Frequent migration, wide distribution of *Anopheles* mosquitoes, and poor treatment contributed to the high incidence and recurrence in Tengchong County [6]. For instance, 2709 cases occurred in 2005, a 34.6 % increase on 2004, mainly due to an arrival of migrant workers from Myanmar during December 2004–April 2005 [7].

Another risk is that malaria in the Sino-Myanmar border areas has not been effectively controlled [8]. Wang conducted a survey in four special regions of northern Myanmar, near China and showed that the average prevalence of malaria infection was 13.6 % [9]. In Kachin State Special Region-1, neighbouring Tengchong County, the main source of local income is grain cultivation, coupled with income from scattered rubber and sugar cane cultivation. Local people live in extreme poverty and education levels are generally low. In these communities, socio-economic under-development, an unstable political situation, relatively weak healthcare infrastructure, and difficult access to healthcare services all provide challenges to implementing and maintaining effective malaria prevention and control. The long China-Myanmar border, which has no natural barriers, is subject to frequent cross-border migration, making it difficult to manage a migrating population through appropriate channels.

In this finding, the malaria epidemiology in Tengchong County was characterized during 2005–2014, an important transition period from control to elimination phase, in order to provide evidence-based proof to support adjustment of appropriate strategies and interventions towards malaria elimination, which could be referenced by other counties along the China-Myanmar border.

## Methods

### Data collection

A retrospective study was conducted to explore malaria-endemic characteristics during 2005–2014 in Tengchong County. All individual cases from a web-based reporting system (WBRS) were carefully reviewed and analysed. The data were selected by onset of date, reporting area and final confirmation. The WBRS parameters consisted of species composition, geographical distribution, gender and age distribution of cases, and number of deaths. Clinically diagnosed cases were defined as a patient with history of travel to an malaria-endemic area and with malaria-like symptoms but no parasites detected by blood examination. Laboratory-confirmed cases were defined as using any of the laboratory tests, including polymerase chain reaction (PCR), rapid diagnostic tests (RDT) and microscopy examination. Both clinical malaria and confirmed malaria cases were included in the present study. For the total number of cases of local infections and imported malaria cases, data were obtained from another system, an annual reporting system. In China, any cases defined as imported malaria must meet all of the following criteria: (1) by giving a diagnosis of malaria; (2) with a travel history to malaria-endemic areas outside China during malaria transmission season; and, (3) the onset time of the patient being less than one month after returning to China during the local transmission season. This definition is based on the reasonable incubation period for all *Plasmodium* species reported in China [10].

### Data analysis

A descriptive analysis was processed using Microsoft Excel and SAS software (SAS Institute Inc, Version 9.2, Cary, NC, USA). Distribution maps of imported malaria in 2005, 2010 and 2014 were created by ArcGIS 10.1 (Environmental Systems Research Institute, Inc, Redlands, CA, USA). Annual parasitic incidence (API) = total confirmed cases in a year × 1000/total population.

## Results

**Fig. 2:**
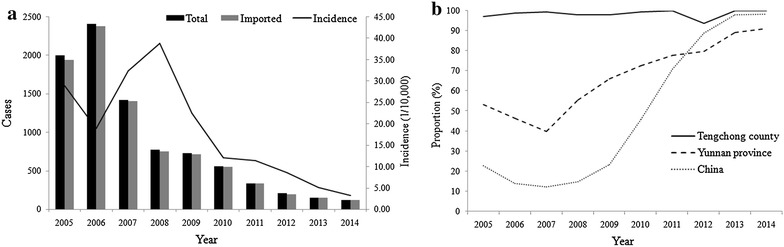
Malaria prevalence in Tengchong County, 2005–2014. **a** The columns show the change trend of total cases (*black*) and those imported from other countries (*grey*), respectively. The *black line* indicates malaria incidence during 2005–2014. **b** The trend of proportion of imported cases in Tengchong County, Yunnan Province and China

A total of 8321 malaria cases were recorded by WBRS during 2005–2014, among whom 7615, accounting for 91.5 % of all cases, were reported from 2005 to 2010 [5, 11–18]. During this period, reported malaria cases reached a peak in 2006 (n = 2206) with the highest incidence (35.4/10,000) (Fig. 2a). Unlike Yunnan Province and the whole country, the proportion of imported cases remained at high degree all the time in Tengchong County, 2005–2014 (Fig. 2b).

**Fig. 3:**
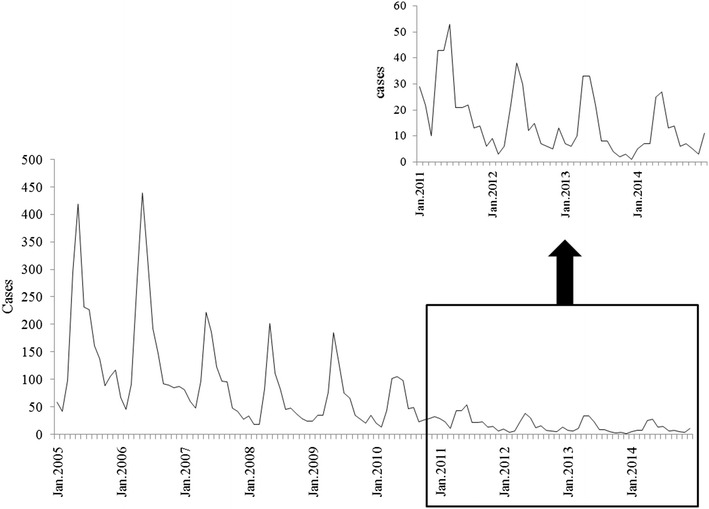
Monthly distributions in Tengchong County, 2005–2014

Cases in Tengchong County declined annually from 2010, when the malaria elimination programme was launched. There was a 76.6 % decrease noted from 2010 (n = 556) to 2014 (n = 130), and no local transmission has been observed since 2013. Malaria cases occurred in males 92.9 % (n = 7733) and 7.1 % in females (n = 588) with similar distribution for all age groups. Most malaria cases were observed in the age group 30–34 years, 94.1 % in males and 5.9 % in females. Cases were mainly reported April–October and December–February, when migrate workers returned for farm work and to enjoy the Spring Festival, respectively (Fig. 3).

In addition, WBRS reported 931 clinically diagnosed (11.2 %) and 7390 laboratory-confirmed cases (88.8 %) from 2005 to 2014, respectively. The proportion of clinically diagnosed cases had decreased since 2006, and since 2013 there were no clinically diagnosed cases reported in Tengchong County. From 2005 to 2014, WBRS reported five malaria deaths (0.06 %, 5/8321), all *Plasmodium falciparum* infections; Jietou, Mazhan, Hehua, Qingshui, and Wuhe reported one death each. The highest number of deaths occurred in 2005 (n = 3).

### *Plasmodium* species

**Fig. 4:**
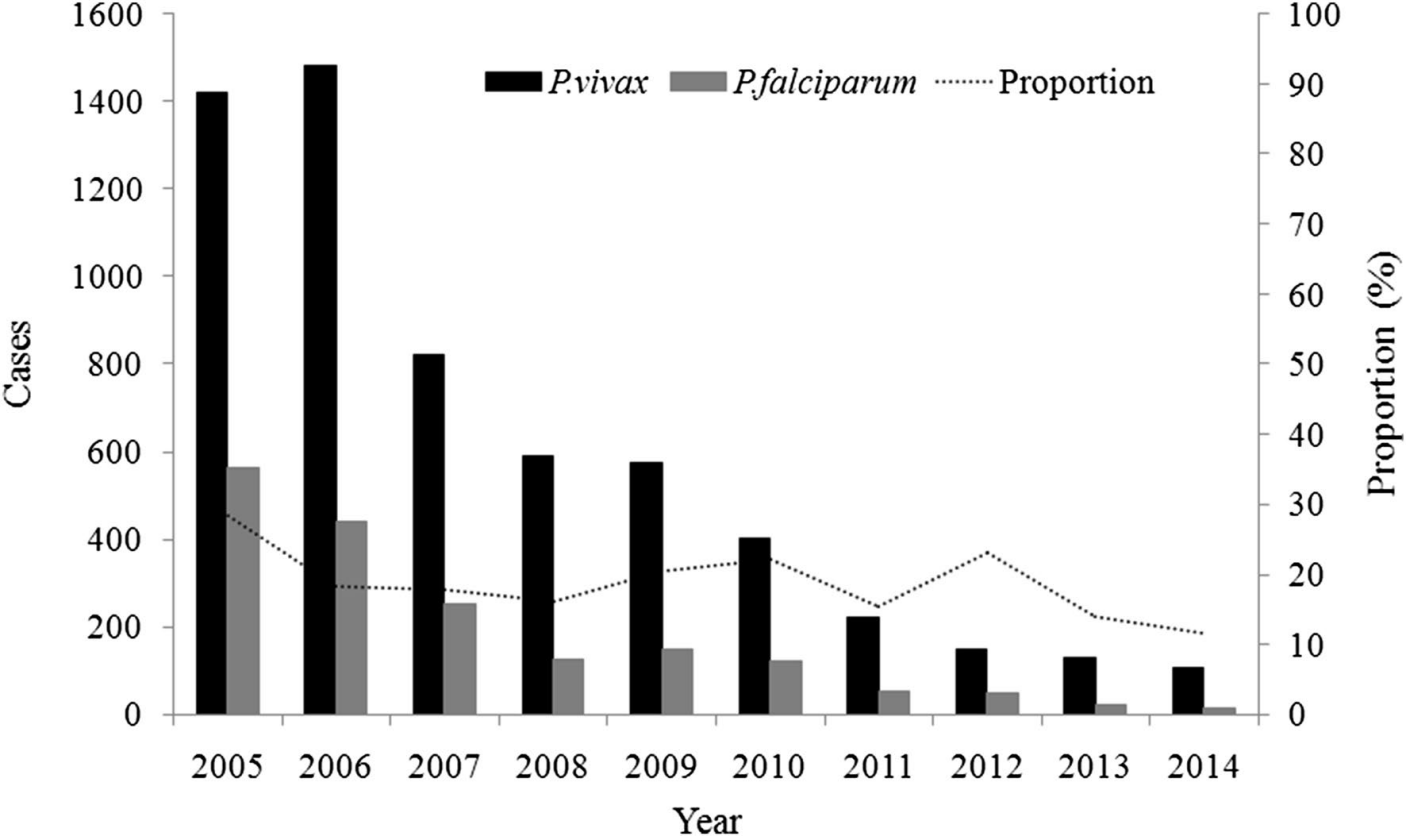
Trend of *Plasmodium vivax* and *Plasmodium falciparum*, Tengchong County, 2005–2014. The *lines of different columns* show the change trend of *P. vivax* (*black*) and *P. falciparum* (*grey*). The *dash line* represents the proportion of *P. falciparum* in the total cases

In total, 5867 *Plasmodium vivax* and 1783 *P. falciparum* cases were recorded in Tengchong County, 2005–2014 (Fig. 4). The proportion of *P. falciparum* gradually reduced from 11.5 % in 2014, down by 59.4 % compared with 2005. All the townships had a higher proportion of *P. vivax* (the mean *P. vivax* to *P. falciparum* = 3.3–1). The highest ratio of *P. vivax* to *P. falciparum* was observed in Puchuan Township with 6.4 and lowest ratio occurred in Heshun Township with 2.3.

### Regional distribution

**Fig. 5:**
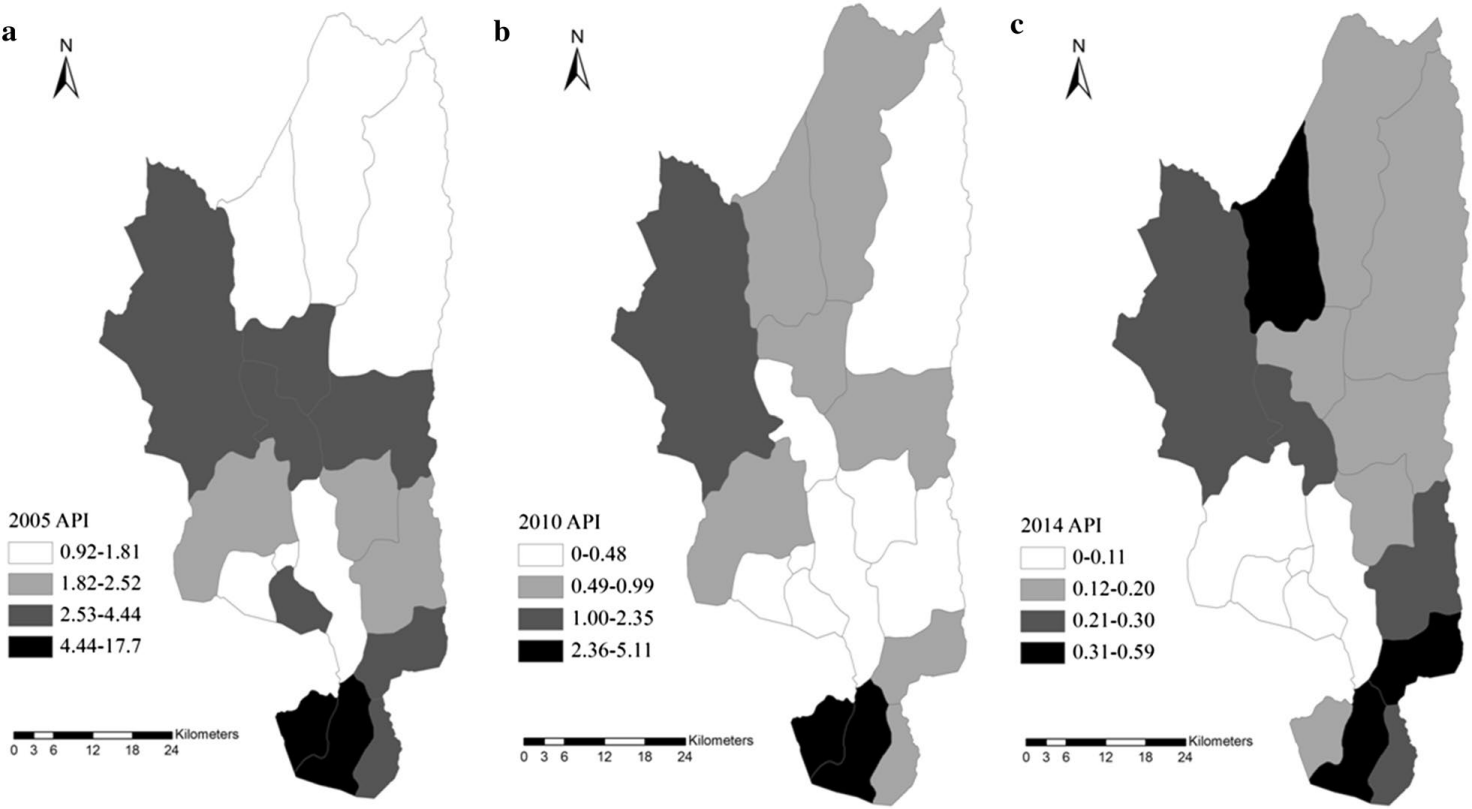
Annual parasite index distribution in Tengchong County, in 2005 (**a**), 2010 (**b**) and 2014 (**c**). Map created by ArcGIS 10.1. The *different coloured squares* indicate reported case number ranges

Malaria cases were mainly distributed in three townships: Wuhe (n = 1542, 18.5 %), Mangbang (n = 1066, 12.8 %) and Gudong (n = 774, 9.3 %). The API for each township decreased gradually. For instance, the highest API was observed in Xinhua Township with 17.7 in 2005, while in 2014 the highest API occurred in Wuhe Township with 0.59. Additionally, the south part was the severe region as the API for this area was almost the highest in Tengchong County from 2005 to 2014 (Fig. 5).

### Source of infection

Data from annual reporting system of 2005–2014 indicated 147 local malaria (1.8 %) and 8174 imported malaria (98.2 %) cases. During this period, local transmission decreased by 77.6 % from 2005 (n = 58) to 6.3 % in 2012 (n = 13), and since 2013 no local cases have occurred. Local cases were mainly reported in the townships of Qushi (n = 29, 19.7 %), Jiehe (n = 18, 12.2 %) and Zhonghe (n = 12, 8.2 %). Local *P. falciparum* (n = 49) occurred mainly in Qushi (n = 17) and Jiehe townships (n = 7). Interestingly, Puchuan reported one local *Plasmodium malariae* in 2009, confirmed both by microscopy and RDT.

Contrary to decreasing local transmission, the proportion of imported malaria has continuously increased from 2005 (97.1 %) to 2014 (100.0 %). Of the WBRS-recorded 757 imported malaria cases during 2011–2014 in Tengchong County, 716 cases (94.6 %) were returned from Myanmar. The cases imported from Myanmar observed a 46.4 % decrease from 2011 (n = 282) to 2014 (n = 112). This was probably because the decrease in malaria cases also occurred in the Myanmar. The World Health Organization (WHO) reported that the number of cases reported in 2013 (333871) decreased by 30.2 % when compared to 2012 (n = 478084) in Myanmar [1, 19].

## Discussion

Tengchong County is one of 18 border counties on the China-Myanmar border classified into Type II counties according to NMEP, with an emphasis on disposal of any possible malaria cases and active foci in order to interrupt local transmissions [4].

Tengchong County had experienced a declined period during 2005–2014. There are several factors that contribute to the decreasing trend in malaria prevalence in Tengchong County, 2005–2014: firstly, Tengchong County obtained the support of the Global Fund Rounds five and six, which provides a financial guarantee and offers help with malaria control. For example, the number of blood detections and insecticide-treated net (ITN) distribution was 25466 and 36093 in 2011, increasing by 105.3 and 409.9 % compared with 2006, respectively; secondly, China launched NMEP and more strict requirements on interventions, including epidemiological survey, vector control, data or sample collection and storing, etc., which facilitates intervention implementation to progress malaria elimination. In 2012, Tengchong County Center for Disease Control and Prevention (CDC) was chosen as the malaria elimination base co-operated by the National Institute of Parasitic Diseases (NIPD), this strengthens technical support and promotes the elimination process; thirdly, the establishment of joint control and prevention mechanism in 2010 facilitated information exchange and communication with Kachin State Special Region-1 of Myanmar and six information exchange meetings were held until 2011; finally, the Chinese Government prohibited illegal logging from Myanmar since 2006, which has largely decreased importation of malaria in patients returning from Myanmar [20].

Another characteristic in Tengchong County is the high proportion of imported cases all the time. Unlike the whole country, importation was more than 95 % all the time in 2005–2014 except 2013, while the proportion of imported cases in Yunnan Province and the whole country gradually increased after 2007 [21]. This was because of the frequent movement of population going to Myanmar for trading, migration and travel. The high proportion of imported cases brings high risk to local facilities, as well as the wide distribution of *Anopheles* mosquitoes, which may cause secondary infection and outbreak epidemic [22]. Therefore, how to identify imported infections from neighbouring countries is crucial and has been a great challenge especially in border regions. WHO recommends the screening for malaria infection at border checkpoints not only to identify and treat malaria cases, but also to set up response measures to prevent re-introduction across the border. More active measures may be needed at border immigration checkpoints, to target longer-term migrant workers [23]. To solve this, Tengchong County has launched four border malaria posts and screens febrile patients for malaria by RDTs and microscopy. The posts also provide an outreach service to nearby villages for active detection using RDT and deliver Information, Education and Communication (IEC) materials.

Combined with importation, the emergence of resistance to artemisinin found on the Thai-Cambodian border has also become a great concern for malaria elimination in Tengchong County [24, 25]. Although artemisinin-based combination therapy (ACT) is generally effective in treating *P. falciparum* in Tengchong County, there has been some indication of reduced sensitivity of parasites to artemisinin and its derivatives, and some mutation of genotype against *P. falciparum* observed along the China-Myanmar border [26, 27]. Rigorous clinical efficacy studies are still warranted in this region due to frequent usage of monotherapy over several decades.

To deal with imported cases, a surveillance system should be established to target mobile and migrant populations and ensure timely recognition, prompt response and effective management. Accurate RDT and PCR confirmation should be developed for efficient surveillance [28]. CDC staff should carry out screening work on high-risk populations of Chinese nationals returning to Tengchong County (in communities) and for Myanmar migrants coming to Tengchong County. What is also needed is for the existing cooperation mechanism to remain, such as joint prevention and control between China and Myanmar, and for the establishment of an information-sharing platform of surveillance and response. Additional steps can be taken to prevent malaria resurgence, including mosquito control measures in the areas with high receptivity and improved accessibility to qualified anti-malarials and compliance of treatment. If these strategies are undertaken it will greatly reduce the risk of malaria re-introduction, and speed up achievement of the goal of malaria elimination in Tengchong County.

## Conclusions

Malaria prevalence in Tengchong County has significantly declined in the past decade. However, the proportion of imported malaria by frequent mobile and migration populations will pose a great threat to the elimination programme. Therefore, surveillance systems need to be carefully planned and well managed to ensure timely recognition and prompt response to imported cases as well as to remove any residual local cases. In addition, effective mechanism of multisectoral coordination and cooperation, joint prevention and control should be strengthened. The health authority should strengthen verification and trace investigation of any malaria cases, including indigenous and imported ones, which are required to ensure elimination of any potential reservoir and prevention of local transmission caused by imported pathogens.
